# Supplementary material for: A Functional Network of Novel Barley MicroRNAs and Their Targets in Response to Drought
Source: Genes (Basel). 2020 Apr 29;11(5):488. doi: 10.3390/genes11050488 (PMC7290300; doi:10.3390/genes11050488)
Supplement: Supplementary file 1 [file genes-11-00488-s001.zip › supplementary/Supplementary data.docx]

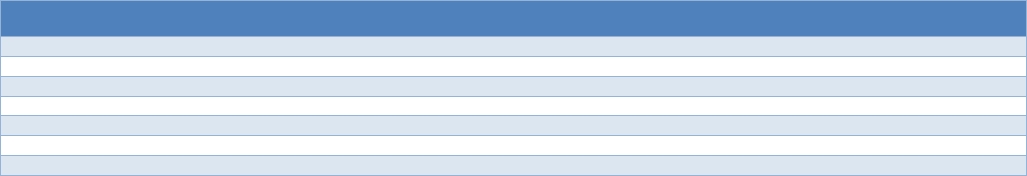


**GTATTCAACCCTGTCAAAGGA**

**AS064**

**3'RACE for miRNAhvu-x13**

**GGTAGGCCGTGAACCAAATCGCCACA**

**AS062**

**5'RACE for miRNAhvu-x13**

**CTGCAAGTCACGGCCATGGGACA**

**AS048**

**3'RACE for miRNAhvu-x10 and miRNAhvu-x8**

**GGAAGCAGCACTCAATCCGATCGAA**

**AS046**

**5'RACE for miRNAhvu-x10 and miRNAhvu-x8**

**CCGTTTCTCCAGACCCCGCTGAC**

**AS050**

**3'RACE for miRNAhvu-x11**

**ACTGTGAGCAAACCATCATCTGCCC**

**AS052**

**5'RACE for miRNAhvu-x11**

**CTCGCTGGACAAGTGCGTCAACA**

**AS054**

**3'RACE for miRNAhvu-x9b**

**miRNA***

**sequence**

**68th day of**

**miRNA hvu-x10**

**cgccatccttggtatctttga**

**1**

**2**

**1**

**1**

**1**

**miRNA hvu-x9b**

**ggagcatgattcaaccaattg**

**13**

**43**

**98**

**46**

**60**

**miRNA hvu-x13**

**aacttgtagaggtgatttggt**

**14082**

**2334**

**2267**

**1434**

**4210**

**miRNA hvu-x8**

**ccatccttggtatctttgatatcg**

**5**

**3**

**8**

**1**

**9**

**Supplementary Table 1. A list of miRNA* for novel barley miRNAs**

**1week 2week 3week 6week development**

**miRNA hvu-x9a gaagcatggttcagccaattg 4 11 14 12 12**

**miRNA hvu-x11 tgaacatcccagagccaccgg 13681 13695 19026 12017 12762**

**miRNA hvu-x5 ggattttgcagatctggcgac 20 25 45 40 56**

**Supplementary Table 2. A list of primers used in the study**

**sequence number**

**CAAATGCAAGGCTGTGTGTGGCAT AS065 3'RACE nested for miRNAhvu-x13**

**CCGGACGAACAAGACAAAACGAAAGC AS063 5'RACE nested for miRNAhvu-x13**

**TGATATCACCAGACACTTGGCCCGT AS049 3'RACE nested for miRNAhvu-x10 and miRNAhvu-x8**

**CAGACTCGACAAGCTGCCTAGGACA AS047 5'RACE nested for miRNAhvu-x10 and miRNAhvu-x8**

**GTCCTCGAGCCCTCCACCTCAGTC AS051 3'RACE nested for miRNAhvu-x11**

**AAGATAACGGGCCTCACCTCCGTTG AS053 5'RACE nested for miRNAhvu-x11**

**TTATGCTTTCACCGTCGATGCCCAC AS055 3'RACE nested for miRNAhvu-x9b**


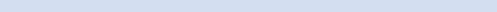


**GTTTCTCCAGACCCCGCTGAC**

**Os.29F**

**forward primer used for amplification of pri-miRNA hvu-x11**


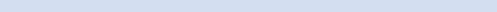


**GGCCGTCTCAATTGACAGGAATGG**

**AS056**

**5'RACE for miRNAhvu-x9b**

**CACCGATTCTAACTGCCATCCACGT**

**AS058**

**3'RACE for miRNAhvu-x9a**

**CATCCCGTGTGATGATGTTGGAGACG**

**AS060**

**5'RACE for miRNAhvu-x9a**

**CAGAAGCATGATCCAACCAGT**

**AS062**

**hybridization probe for miRNAhvu-9a**

**CCGGTGGCTCTGGGATGTTCA**

**AS064**

**hybridization probe for miRNAhvu-11**

**GTCGTCACCCCTCAGATCTTT**

**AS066**

**hybridization probe for miRNAhvu-8**

**GGATACTTGAAGGATTCGTTGCT**

**F57686.2**

**forward primer flanking miRNA recognition site in MLOC_57686.2**

**TGCCAAGCTGTGCATCTCTGTG**

**F56533.1**

**forward primer flanking miRNA recognition site in MLOC_56533.1**

**CTCAACGAGACAGTGGTGGT**

**F80127.2**

**forward primer flanking miRNA recognition site in MLOC_80127.2**

**CTCTCGCCTCAGTGATCGAC**

**F601197.2**

**forward primer flanking miRNA recognition site in MLOC_601197.2**

**GTGCATGTGATGTGAGCGTC**

**F26843.2**

**forward primer flanking miRNA recognition site in MLOC_26843.2**

**ATGGGATGAAGTGAAGAGTGC**

**Os.9.1F**

**forward primer used for amplification of pri-miRNA hvu-x9a**

**ATGTGGTACCTTGTATGGGATGA**

**Os.15F**

**forward primer used for amplification of pri-miRNA hvu-x9b**

**AGCATCTCAATTCCAGCAAGT**

**Os.13F**

**forward primer used for amplification of pri-miRNA hvu-x8 and pri-miRNA hvu-x10**

**GGTGCAGCATGTGTGGCTTGTACTT AS057 5'RACE nested for miRNAhvu-x9b**

**GGAGCGACCCTGCCTGACATCCC AS059 3'RACE nested for miRNAhvu-x9a**

**CCTTCTCCTTCCTGGGCTCTGGTTG AS061 5'RACE nested for miRNAhvu-x9a**

**CAGAAGTATGATCCAGCCAGT AS063 hybridization probe for miRNAhvu-9b**

**CACCCCTCAGATCTTTGATAT AS065 hybridization probe for miRNAhvu-10**

**ACCAAATCACCTCTACAAGTT AS068 hybridization probe for miRNAhvu-13**

**TGCACCATCACATGAACCTGA R57686.2 reverse primer flanking miRNA recognition site in MLOC_57686.2**

**TCACCTCAACGTATTCTTCAAGT R56533.1 reverse primer flanking miRNA recognition site in MLOC_56533.1**

**GCAAGTCACTCCTACATGCG R80127.2 reverse primer flanking miRNA recognition site in MLOC_80127.2**

**TGGCTCAGTCACAAGCAGAG R601197.2 reverse primer flanking miRNA recognition site in MLOC_601197.2**

**ACGACGCGGGAGTAGTTAAT R26843.2 reverse primer flanking miRNA recognition site in MLOC_26843.2**

**GGGCCAAAATTTCAAACTGGG Os.9.R revers primer used for amplification of pri-miRNA hvu-x9a**

**AATCAATCACCATATCATGTA Os.15.R reverse primer used for amplification of pri-miRNA hvu-x9b**

**ATGGGGGGCATAGGGAAGATG Os.13R revers primer used for amplification of pri-miRNA hvu-x8 and pri-miRNA hvu-x10**

**GAAAAAGCAATTCAAGCATCC**

**Os32F**

**forward primer used for amplification of pri-miRNA hvu-x13**

**AGGCCAACAAATCCATTAAAT Os29R revers primer used for amplification of pri-miRNA hvu-x11**

**AGCATTGAAGGTGCTTGGCAT Os32R revers primer used for amplification of pri-miRNA hvu-x11**

**Supplementary data 3. Sequences of obtained pri-miRNAs**

**Pri-miRNA hvu-x9a**

ATGGGATGAAGTGAAGAGTGCCGAGTGTCTTGATCTTACTAATCAATGCATCAAATTATGTGCACAATAAGCTTTTGCTATGCTCTCACCGTTAATGTCCACT

GGTTGGATCATGCTTCTGTTTATGAGCTCATTGAAGTAATTCTCTCGTAGCTCAAATTGGCCAATTTCTCGTTTGCGTGGCACACAAAACCTTCAGCTATCCAT

CTCCATATCTGTCGGATCTCTCTTAATTTTGTAATCTTCTGGAAATATGCTTAGGTGTAATAAACGATGCGAAGGTAGATCATAAAACATGCACCAATACTAT

GTGAAATATCAAAGAAAAAAAGTAAAAGAGGCCCTTGACTTGTAGATTATACAAGAGTTATGATCTAGAAGTCGACCCTTTTTTCCTAGTAGTTCTGATCTA

CCTTCCCATCTAATAGGATTAGTTATTATGTCTAAGCATGTTTTTAGAAGATTAGATGATTAAGCAAGCTCATTTGATATGTAAGTGGATAGTTTGAGGTTTTG

TTTATCATGAAAAACAAGGGATTAGCAAGTTTGAGCTCGGTGGTAGAATTGCTCAATGAGACCGTGAATAGGAGCATGATTCAACCAATTGACATCAATGTT

GGTAAATGCATGGAAAAATCTTATTGTATACATGATATGGTGATTGATTCCATATGTTTCTGCCAAGTGAAGAAAAGTCACTTCAATTGAAGCGATAACGGT

CTGTCCAAAAGCAAGATTTGAAGATTATCCATCCCAAAGTGCATGACAGGTAGCAGCTATGCCACAAGTCAGGTCTCTTGCAATTGTTGATTCATTGTAGCTT

ATGCTGAGTGTTCAGTTCTACATGTTTGTCTGTCCTTTGCAATCAGGATGATTCAAGAACATTGCATTTGTCTTTGTTTTGATTTTTCTTCCTTACGCAATGACA

TGCAGCTCTCATTGTATCTACCCACTTATGCTGAATTGTATTGTCATGAAACTTTTAGTTGTAGCCACAGTTTTGGATTTCGTCCGGAAAAAACGAAATTCCAG

CGAAATCCGCCCGTTTCGTTCAGGCCCGGTAGATTTGTTTACCGGTCAAAAAATTCGGCCCAGTTTGAAATTTTGGCCCAAAAAAAAAAAAAAA

**Pri-miRNA hvu-x9b**

ATGTGGTACCTTGTATGGGATGAAGTGAAGGCTATTTTGTTAATCAATCATCATATTTTAATCAATCATCATATTATGTGCACAATAAGCTTTTGTTATGCTTT

CACCGTCGATGCCCACTGGCTGGATCATACTTCTGTTTATGAGCTCATTGAAGTAACTCTCTCCTAGCTTAAATCGGCCAATTTCTCGTTTGCATTATATATAA

AACCTTCAGCTATCCATCTCCATATCAGTTCGTCTCTCTTAATTTTGTAATCTTTTGGAAATATGCTTAAATATCCTCAAACATAAGTACAAGCCACACATGCT

GCACCAATACTATGCAAAGTAGCATAGAGTGAGTAAAATAGGCTCTTGACTTTTAGATTATACAAGAGCGGTTATCTACAAGTCAACCCTTTTTTTACTAACT

ACTAGTAGCCTTGGTATATCTATATGATTAGTTATTATGTCCAAGTATGTTTTTAGAAGATTAAGCAAACTCATTTGATATGTAAATGGATAGTTTGAGGTTTT

ATTTATAATGATAAACAAGGGATTAGCAAGTTTGAGCTCGGTGATAGAATTACTCAATGAGATCGCAAATAGAAGCATGGTTCAGCCAATTGACATCAATGT

TGATAAAGGCATGGAAAAATCTTATCGTATACATGATATGGTGATTGATTAAAAAAAAAAA

**Pri-miRNA hvu-x8 and pri-miRNA hvu-x10**

AGCATCTCAATTCCAGCAAGTTCGAGTGGGTAACGAGTATTCACCAACAGTGAAAAAGATCATNCCGTGAGAGTCCTCAACCAGCGCATGTGAACAAGCAG

CGCATGCCGCAACCATGGGACAACGCCGCCATCCTTGGTATCTTTGATATCGCCGGACAGTTGACCCGCTCAGATGGCAGACTCGACAAGCTGCCTAGGACA

CCCGTCCATTGCAAGCCAAACTTTAAGGCCGTGAAGACAGCCATAGAAATGTATACCGCCTTGGAGGGGTCAACGCTGAACATGTCTAAGGCTGTTTTCATG

GCCTGAAAGTTTGGTTTGCAGTGCACAGGGGTCTTAGGCAGCATGTCGACCTTGCCACCTGAACGGGCCAAGTGTCTGGTGATATCAAAGATCTGAGGGGTG

ACGACCTTGTCCCATGGCCGTGACTTGCACGACTTGTTTTGAGTCTCGAAGCAGATGATCCCATTCCAAAGCCCATCTTCCCTATGCCCCCCATAAAAAAA

**Pri-miRNA hvu-x11**

GTTTCTCCAGACCCCGCTGACGACGCCGCTCACCACCTTCCGCGGCTACCCCCGTCCTCGAGCCCTCCACCTCAGTCGGGCCGTGTCTCCTTCTGCTGGATCGC

GTCCGGATGACGGGCGCCGTCTGCCGGTTTTGTCGCGGAGCCCGGCGGCTCTGTGGTGTTCAAGCAGGAACCTCATGCTACCGGCAGCATGCGGCGCTTGCT

TGAACATCCCAGAGCCACCGGCGTGCCAAAATTCACGCAGAAGGAGATCGATCTCTCGAGGAGAGATCCGCCGCCAAGCTGGAGTACGTGAACGTCTTCTTC

AAGTGCTTCACTTTTTTCTTTTCTTGCGGAGAACCCCCAATACCGTGTTTATCCTCAACGGAGGTGAGGCCCGTTATCTTCAATTCTGCATTGTAGGCTGTTGT

ATCCTCATTGTGTTTTATGTTTTGCTGCCGGTGCATATGATAAGTATATGCTGTATGCACATACTAGCTTGTTATTCAGTGTAATACTAGCTTGTTATTCAGTGT

AGGCATGTGCGTTCGGGCAGATGATGGTTTGCTCACAGTGTGCGTTCTAAAACGAACATTTATAATTTAATGGATTTGTTGGCCTAAAAAAAAAAA

**Pri-miRNA hvu-x13**

GAAAAAGCAATTCAAGCATCCAAGCAGTAGTACATGAGATAGCTTCTCAAATGCAAGGCTGTGTGTGGCATGTGTGTGCAGCCTCTCTCTGATCCTCTTTGTT

CCGGGTGAAATGAAAACTTGTAGAGGTGATTTGGTGATCACCGAATCTCTTGTATTCGGTGCACACCAAATCACTTCCACATGCTTTCGTTTTGTCCTGTTCGT

CCGGTATGGACCGTATGGTATGCCTCGTCATGAGGCGTATCTACACACGGCTATCAAGGATGGCCGTGTGTGGCGATTTGGTTCACGGCCTACCACACATTA

AGCACGCGGACGAGCGAGCTCTATGAGTCGTGGCTCGTCGGGAAGCAACGCCATCGATCGCCAAATTAACAAGCTCCAAAAGCAATTTCAGAACTATTGTAT

ACTTGTTTCAGGTAGAACACATCAAAAAGAGGTGACCAAAAGGTAAAGATCATACTACGTTAATCAAGTAATATTGTTATCATAATGGAGTGTCAATGCCAA

GCACCTTCAATGCTAAAAAAAAAAAAAAAAAAAAAAAAAAAAAAAA


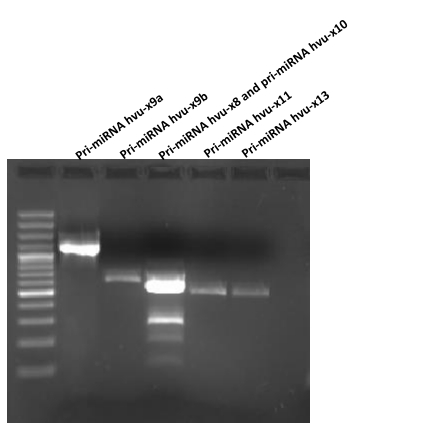


**Supplementary data 4: Agarose gel showing amplified pri-miRNAs**

**100bp**

**plus**

*

*Unspecific product


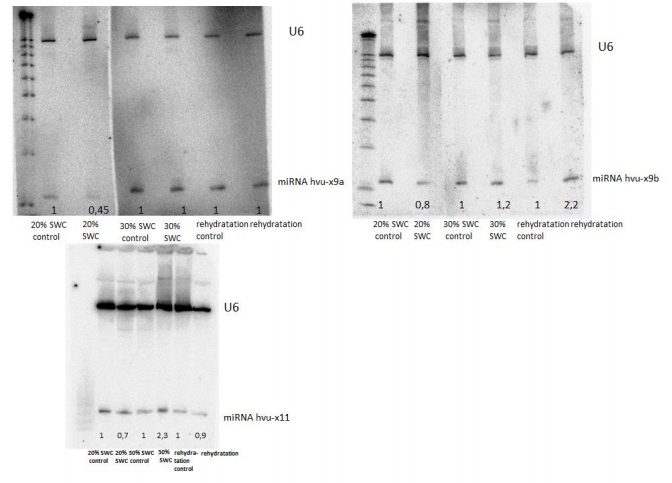


**Supplementary data 7: Northern hybridization of miRNAs in drought conditions**

***wide blots were cut to the size of hybridization bottles and reassembled to exposition**
